# Modeling and mapping the current and future distribution of *Pseudomonas syringae pv*. *actinidiae* under climate change in China

**DOI:** 10.1371/journal.pone.0192153

**Published:** 2018-02-01

**Authors:** Rulin Wang, Qing Li, Shisong He, Yuan Liu, Mingtian Wang, Gan Jiang

**Affiliations:** 1 College of Agronomy, Sichuan Agricultural University, Chengdu, Sichuan, China; 2 Sichuan Provincial Rural Economic Information Center, Chengdu, Sichuan, China; 3 The Kiwifruit Institute of Cangxi Country, Cangxi, Sichuan, China; 4 Sichuan Meteorological Observatory, Chengdu, Sichuan, China; University of the West of England, UNITED KINGDOM

## Abstract

**Objective:**

Bacterial canker of kiwifruit caused by *Pseudomonas syringae pv*. *actinidiae* (Psa) is a major threat to the kiwifruit industry throughout the world and accounts for substantial economic losses in China. The aim of the present study was to test and explore the possibility of using MaxEnt (maximum entropy models) to predict and analyze the future large-scale distribution of Psa in China.

**Method:**

Based on the current environmental factors, three future climate scenarios, which were suggested by the fifth IPCC report, and the current distribution sites of Psa, MaxEnt combined with ArcGIS was applied to predict the potential suitable areas and the changing trend of Psa in China. The jackknife test and correlation analysis were used to choose dominant climatic factors. The receiver operating characteristic curve (ROC) drawn by MaxEnt was used to evaluate the accuracy of the simulation.

**Result:**

The results showed that under current climatic conditions, the area from latitude 25° to 36°N and from longitude 101° to 122°E is the primary potential suitable area of Psa in China. The highly suitable area (with suitability between 66 and 100) was mainly concentrated in Northeast Sichuan, South Shaanxi, most of Chongqing, West Hubei and Southwest Gansu and occupied 4.94% of land in China. Under different future emission scenarios, both the areas and the centers of the suitable areas all showed differences compared with the current situation. Four climatic variables, i.e., maximum April temperature (19%), mean temperature of the coldest quarter (14%), precipitation in May (11.5%) and minimum temperature in October (10.8%), had the largest impact on the distribution of Psa.

**Conclusion:**

The MaxEnt model is potentially useful for forecasting the future adaptive distribution of Psa under climate change, and it provides important guidance for comprehensive management.

## Introduction

Kiwifruit, Actinidiaceae, *Actinidia* Lindl., is a type of perennial, deciduous woody liana and an important class of berry fruit. There are sixty-six different species in the Actinidiaceae family, sixty-two of which originated in China [1, 2]. “Hayward” is the most popular variety of kiwifruit grown across the world, and it was selected in New Zealand from seeds coming from Yichang City, Hubei Province in 1904. As a rich nutritional source of sugar, protein, amino acids and vitamins, and an especially high vitamin C content, the kiwifruit is known as “the king of the fruit” and has good market prospects. The main areas of kiwifruit production in China are in the northern foot of the Qinling Mountains in Shaanxi Province, the Dabie Mountain area in Anhui Province, Heping County in Guangdong Province, Guizhou Plateau, western part of Hunan Province, and Northwest Sichuan Province. Worldwide, kiwifruit is economically very important, and production reached 3.26 million tonnes per year as of 2013. China is the largest kiwifruit producer, with more than 1.77 million tonnes per year, placing China ahead of Italy. The kiwifruit industry, which developed quickly in recent years, has become one of the specialty industries in China that promotes agricultural development [3, 4].

Bacterial canker of kiwifruit caused by *Pseudomonas syringae pv*. *actinidiae* (Psa) is a destructive disease of kiwifruit that causes great losses in kiwifruit production. The disease not only causes great economic losses but also increases the plant mortality of kiwifruit, and to date, there has not been an effective control measure identified [5, 6]. The symptoms and the infection cycle of bacterial canker are consistent throughout the world [7–9]. Symptoms usually appear in the spring or autumn under climatic conditions that are suitable for disease development. The canker spots can occur on twigs, trunks, leaves and flowers. It first appears as a watery lesion on twigs and trunks, then expands into a white exudation, and eventually turns into a rusty-red profuse exudation [10]. Affected branches necrose, water and nutrient transport are restricted, eventually causing the plant organs to wither and die. The symptoms on the leaves began to appear in early April, when the chlorosis transitions from pale brown to tan; it eventually becomes dark-brown with a surrounding yellow halo and finally wilts and curls. When the buds are infected, the growth rate slows or even prevents flowering, which results in fruit drop or the formation of deformed fruit [11].

In 1984, Psa was first isolated from ‘Hayward’ (a type of *Actinidia deliciosa* with green flesh) in Japan [12]. Currently, the pathogen is widely distributed in the major producing countries of kiwifruit, including China, New Zealand, Italy, South Korea, Iran, France, Portugal, Chile, Spain, Switzerland and Australia, as well as in other countries [13, 14]. In China, the kiwifruit bacterial canker was first discovered in the Dongshan Peak farm of Hunan Province in 1985 [15]. The disease quickly spread to the provinces of Sichuan, Anhui, Hunan and Shaanxi[16]. In Japan and Korea, outbreaks of kiwifruit bacterial canker have mainly affected the ‘Hayward’ cultivar. However, in China and other countries, the disease was highly destructive on cultivars of both *A*. *deliciosa* and *A*. *chinensis*. In Shaanxi Province in 2012, an outbreak of kiwifruit canker was recorded in a red-fleshed cultivar of ‘Hongyang’ and in a green-fleshed kiwifruit cultivar of ‘Xuxiang’. The percentage of trees impacted by the disease ranged from 20% to 70%, even up to 100% in some places [17]. An investigation into the bacterial canker of kiwifruit in Sichuan Province between 2014 and 2016 showed that the disease had common occurrence patterns in different production areas, and the situation is worsening as growing areas expand.

Presently, due to the economic losses and the great destruction to the industry, kiwifruit bacterial canker is considered as the major threat for the cultivation of kiwifruit around the world. As a highly infectious disease, its pathogen (*Pseudomonas syringae pv*. *actinidiae*) has been listed on the A2 List of the Mediterranean Plant Protection Organization (EPPO) [18]. In 1996, the State Forestry Administration of China included Psa on the quarantine list of nationwide objects of forest plants. In 2009, China’s General Administration of Quality Supervision issued a new document on the relevant requirements for prohibiting the entry of Psa into China [19].

The study of suitable habitat is an important field of ecology, and the species distribution model (SDM) has evolved and become an important tool for studying the suitability of a habitat for a particular species [20]. The SDM assumes a species niche should be conserved over space and time, assesses the potential geographical distribution of a target species based on presence/absence data and uses the corresponding mathematical variables to determine habitat preferences for a species. At present, SDMs are mainly applied and influence the following aspects: research on species’ potential geographical distribution, analysis of the relationship between species distribution and climate change, prediction of the habitat suitability of endangered species, and the study of paleogeography [21]. An SDM model, i.e., the maximum entropy model (MaxEnt), has many advantages, including short running time, easy operation, small sample size and high simulation precision, and was applied to simulate the suitable geographical distribution of species suitability [22–24]. In recent years, many researchers have used MaxEnt to simulate the distribution of many plant diseases, such as citrus huanglongbing (caused by *Candidatus liberibacter*) [25], maize downy mildew (caused by *Peronosclerospora maydis*) [26], wheat blast (caused by *Magnaporhe grisea*) [27], South American leaf blight (caused by *Microcyclus ulei*) [28], and pine wilt disease (caused by *Bursaphelenchus xylophilus*) [29]. MaxEnt performs well in this type of application and is widely accepted by ecologists.

Climate change has greatly influenced the distribution of various species, and future climate change will change the habitat, range, and distribution of many species [30–32]. Plant disease is one of the most serious biological disasters to impact agricultural production and is constrained by climate change, host plants, tillage management and farming systems [33–35]. The distribution and abundance of plant diseases are highly influenced by climatic factors (i.e., temperature, moisture, humidity and their seasonal variations) [36, 37]. Temperature is one of the most influential environmental factors affecting the distribution and abundance of different species [38, 39]. In the context of global climate change, where trends indicate increasing temperatures, variations in precipitation and more frequent and extreme weather events have occurred. Additionally, the environment has changed, which has resulted in changes in the areas and periods of plant diseases and led to changes in distribution, occurrence, epidemiology and population structure [40, 41]. SDM is an effective tool for studying the impact of future climate change on species distribution and provides a variety of realistic scenarios to expound the influence of climatic factors on the epidemiological traits of pathogens. SDMs utilize a series of greenhouse gas emission scenarios, which are based on global climate models (GCMs), to analyze the influence of climate change on current and future habitat suitability of various species [42]. Accurate predictions regarding the future state of species will not be provided in SDM at any given point in time, but the possible niche that species may occupy in the future is provided [43].

Present studies of Psa are mainly focused on species classification [7, 44], molecular biology [45–47], analysis of biological characteristics [48, 49], pathogenicity differentiation[50–52], rapid detection methods [48, 53, 54], and disease control [55, 56]; however, systemic research about the influence of climate change on the niches specific to Psa is lacking. In an effort to analyze the effects of climate change on the potential distribution of Psa, MaxEnt was utilized to model the current niches of Psa in China, as well as the future Psa niches under climate change scenarios; additionally, this study identified climatic variables important for the potential establishment of Psa. These results can provide an important reference and theoretical basis for the development of reasonable prevention and control measures.

## Materials and methods

### Occurrence records of Psa

In this study, the occurrence points of Psa were obtained from field data collected by the authors in the Chinese provinces of Sichuan and Shaanxi, from the published literature, and from the online databases GBIF and EPPO (S1 Table). When coordinates were published, we used the records directly. If there were only localities, Google Earth was used to collect coordinates of the records. All occurrence records were checked for accuracy in ArcGIS prior to use. Records with obvious geocoding errors were discarded, and duplicate records were removed manually. All records were imported into Microsoft Excel and saved as “*.CSV” format.

### Environmental variables

From the WorldClim database (http://www.worldclim.org), we obtained 67 environmental variables (19 bioclimatic variables and 48 monthly averages of temperature and precipitation) for the current period [57, 58]. In the Worldclim database, ‘current period’ was defined from 1950 to 2000, and these data have been widely used in creating species distribution models. In 2013, the Fifth Assessment Report was released by the UN’s Intergovernmental Panel on Climate Change (IPCC), and four representative concentration pathways (RCPs, including RCP2.6, RCP4.5, RCP6.0 and RCP8.5) were published in the report [59, 60]. The impacts of climate change strategies on greenhouse gas emissions are considered more in the RCPs scenarios, and the projection of future climate change is more scientifically described. RCP4.5 and RCP6.0 are medium greenhouse gas emission scenarios, and RCP4.5 is of higher priority than RCP6.0 [61, 62]. Therefore, RCP2.6 (the minimum greenhouse gas emission scenario), RCP4.5 (the medium greenhouse gas emission scenario) and RCP8.5 (the maximum greenhouse gas emission scenario) for the 2030s (2021–2040), 2050s (2041–2060), 2070s (2061–2080) and 2080s (2071–2090) were selected for the future model prediction of Psa in China. The future environmental variables were downloaded from the Climate Change, Agriculture and Food Security (CCAFS) website. All environmental variables were in raster format with a 2.5-arc minute resolution (~4.5 km^2^).

Environmental variables derived from WorldClim and CCAFS, which has been widely used in the prediction of the potential distribution of species, can reflect the characteristics of temperature and precipitation as well as their seasonal variation characteristics. The 19 bioclimatic variables with strong biological significance explained the adaptation of species with extreme environmental factors. These variables were also suitable for describing the distribution of species across large scales such as the intercontinental scale [63, 64]. Due to the various reasons mentioned above, the environmental variables provided above were chosen as the initial variables to be used in the modeling in this article. Based on Worthington’s [65] method on how to filter available variables for modeling, the jackknife test was used to evaluate each variable’s contribution to the simulation, and 25 variables were removed due to their lack of contribution (percent contribution = 0). Next, the highly correlated variables were eliminated, and variables with a Pearson’s |r|≤0.8 were retained. After this process, 22 variables (S2 Table) were retained to simulate the current and future distributions of Psa in China.

### Distribution modeling

MaxEnt software was utilized to predict the suitable habitat distribution of Psa in China [66]. MaxEnt uses presence-only and small sample size data to model habitat suitability as a function of environmental variables, and it is consistently among the highest performing SDM methods [67]. Response curves indicate the relationships between climatic variables, and the predicted probability of the presence of Psa was determined by MaxEnt. The percent contribution and permutation importance of environmental variables were calculated, and jackknife procedures were executed in MaxEnt. These analysis methods are all useful to measure the importance of the environmental variables. There were 10 replicates, and a random test percentage was chosen for each replicate. The remaining model values were set to default values [68–71].

MaxEnt estimates the probability a species will be present based on presence records and randomly generates background points by finding the maximum entropy distribution. An estimate of habitat suitability for a species was exported from MaxEnt, and its range generally varied from 0 (lowest) to 1 (highest). Model predictions were imported into a geographic information system (GIS), and maps were generated using ArcMap. Four arbitrary categories of habitat suitability for Psa were defined as no suitability (0–5), low suitability (5–33), medium suitability (33–66) and high suitability (66–100) based on predicted habitat suitability.

In this study, the ROC curve method was utilized to assess the model’s explanatory power [72]. The AUC (area under roc curve) is an effective threshold-independent index that can evaluate a model’s ability to discriminate presence from absence (or background). The evaluation criterion of AUC is illustrated in S3 Table [63].

For reducing the bias of estimation, in 1949, Quenouille [73] proposed an unbiased method of nonparametric estimation, and Tukey renamed it jackknife in 1958 [74]. This method can estimate parameters and adjust the deviation without assumptions of distribution probability. In SDM, the jackknife method was used to analyze the effects of environmental variables on model results to choose dominant factors. The specific process involves 1. Calculating the training gain for the model with only variable. Higher training gain indicates that the variable has high prediction power and contributes greatly to species distribution; 2. Calculating the training gain for the model without a specific variable and analyzing the correlation between the removed variable and the omission error. If the removal of an environmental variable leads to a significant increase in the omission error, it indicates that the variable has a significant effect on the model’s prediction; 3. Calculating the training gain for the model with all variables [68].

### Models of the mean center of highly suitable areas

The mean centers of highly suitable areas of Psa in China were calculated according to Yue’s [75] formula:

$$\left\{ \begin{array}{l} x(t)=\sum_{i=1}^{I}\frac{s_{i}(t)•X_{i}(t)}{S(t)}\\ y(t)=\sum_{i=1}^{I}\frac{s_{i}(t)•Y_{i}(t)}{S(t)} \end{array}\right.$$

In this formula, *t* is the variable of time (i.e., current, 2030s, 2050s, 2070s and 2080s), *I* is the patch number of highly suitable areas, *Si(t)* is the area of *i*th patch of highly suitable areas, *S(t)* is the total area of highly suitable areas, (*Xi(t)*, *Yi(t)*) are the longitudinal and latitudinal coordinate, respectively, of the geometric center of the *i*th patch of highly suitable areas, and (*x(t)*, *y(t)*) are the mean centers of the highly suitable areas. The shift in distance and direction of highly suitable areas in the period from *t* to *t* + 1 are, respectively, formulated as Yue [75],

$$D=\sqrt{{\left(x(t+1)-x(t)\right)}^{2}+{\left(y(t+1)-y(t)\right)}^{2}}$$

$$\theta =\mathrm{arctg}(\frac{y(t+1)-y(t)}{x(t+1)-x(t)})$$

where *D* is the shift in distance of the highly suitable area during the period of *t* to *t*+1; *θ* is the shift in direction of the highly suitable areas, where east is defined as 0°, north is defined as 90°, west is defined as 180° and south is defined as 270°.

Describe the same contents as “Materials and methods” sections with step-by-step protocol on my protocols.io: http://dx.doi.org/10.17504/protocols.io.mdic24e

## Results

### Model performance and contributions of variables

**Fig 1:**
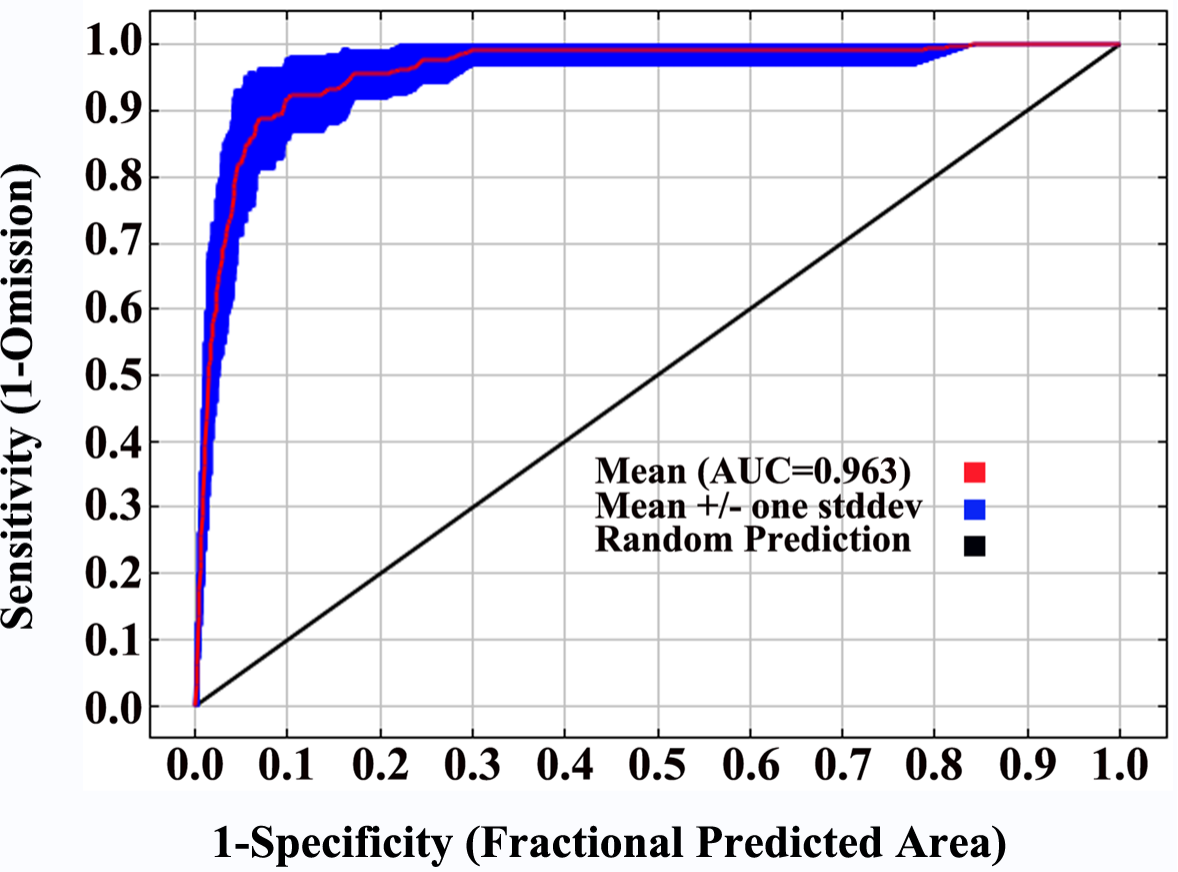
ROC curve and AUC value under the current period (10 runs). The current period is from 1950 to 2000.

In this study, from the ROC curves, AUC values were used to evaluate the performance of the MaxEnt model. Many studies showed that an AUC of high values led to better results that significantly differed from the random predictions. The accuracy of prediction of Psa during the current period was found to be “excellent” (AUC_mean_ = 0.963, Fig 1) according to the identified evaluation criteria (S3 Table).

**Fig 2:**
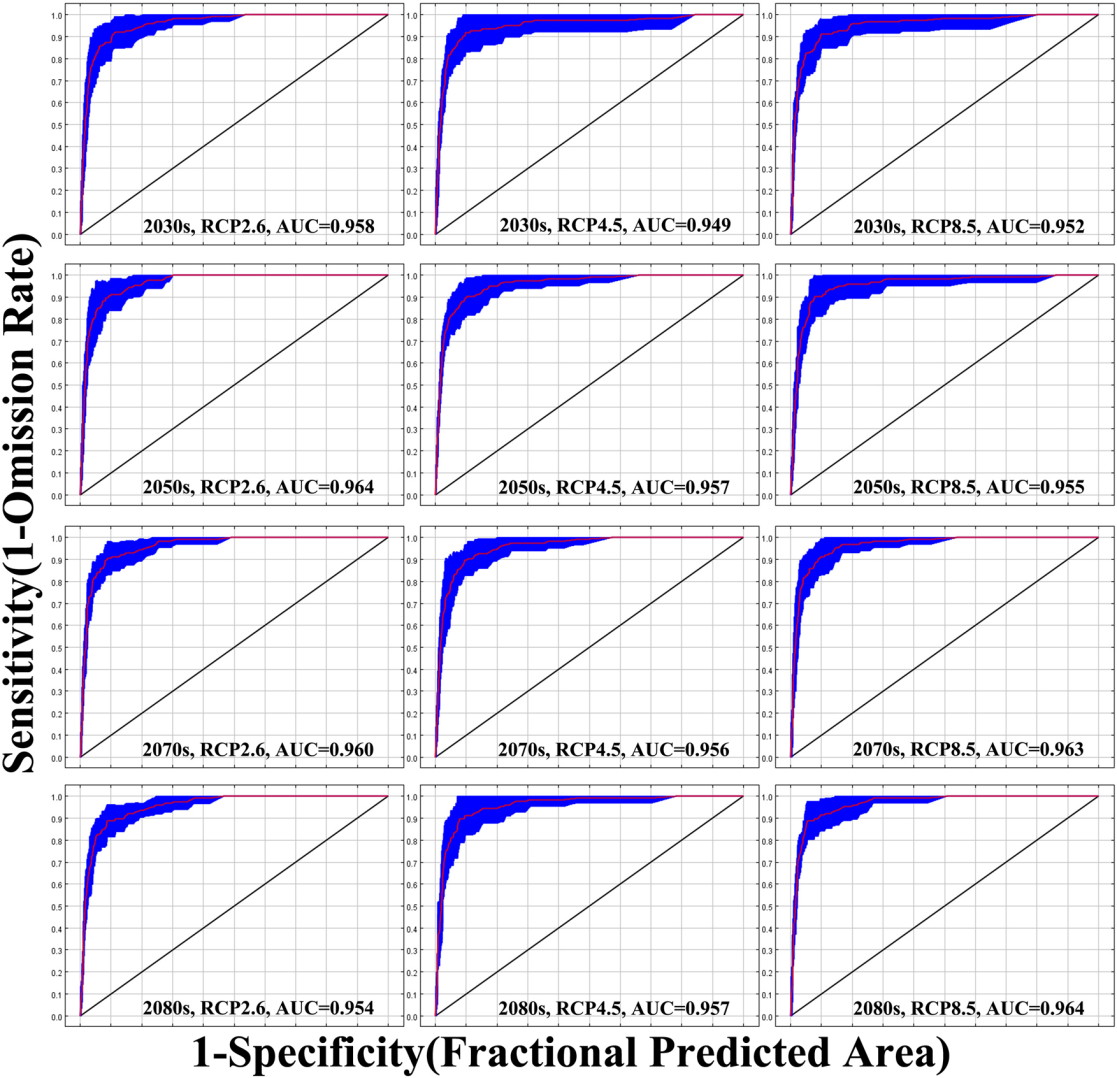
ROC curves and AUC values in future periods. The future periods is 2030s (2021–2040), 2050s (2041–2060), 2070s (2061–2080) and 2080s (2071–2090).

Fig 2 shows that the MaxEnt models that predicted the distribution of Psa in the future period performed “excellent”, with high AUC values (0.949–0.964). The results indicate that the simulations have high reliability and can be used to analyze the impact of climate change on the distribution of Psa in China.

**Fig 3:**
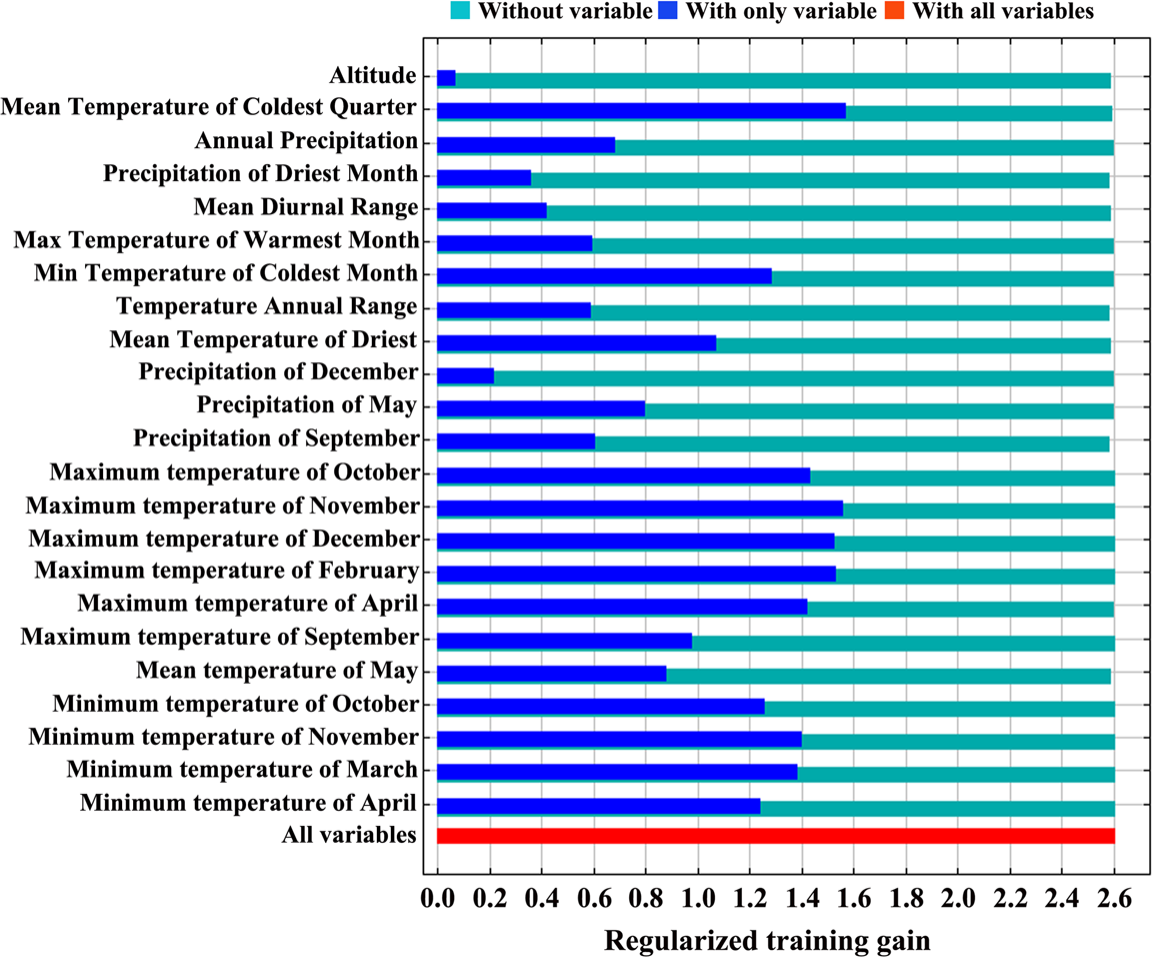
Jackknife test for variable importance of Psa habitat suitability distribution. Values shown are averages over 10 replicate runs.

Among the environmental variables, the maximum temperature in April (19%), mean temperature of coldest quarter (14%), precipitation in May (11.5%) and minimum temperature in October (10.8%) played major roles in the spread of Psa (Table 1 and Fig 3) and individually contributed more to run the model. The other 18 environmental variables, including temperature (maximum temperature in September, October, November, and December; minimum temperature in March, April, and November; mean temperature in May; mean diurnal range, max temperature of the warmest month, min temperature of the coldest month, annual temperature range, mean temperature of the driest quarter), annual precipitation (precipitation in September and December, annual precipitation, precipitation of the driest month) and altitude, individually contributed less (a combined total contribution of 44.7%) to run the model. Considering the importance of permutation, the mean temperature in May (21.8%), mean temperature of coldest quarter (14.8%) and mean diurnal range (10%) each played a vital role in predicting the probable distribution of Psa, and individually, they contributed more than the other variables to run the model.

**Table 1:** Estimates of contribution and permutation importance of environmental variables in MaxEnt modeling of Psa.

Variables		Percent contribution	Permutation importance
**Maximum temperature in April (°C)**	19		2.3
**Mean temperature of the coldest quarter (°C)**	14		14.8
**Precipitation in May (mm)**	11.5		2.6
**Minimum temperature in October (°C)**	10.8		1
**Maximum temperature in October (°C)**	8.7		0.5
**Precipitation in September (mm)**	6.4		5.4
**Mean diurnal range (°C)**	5.7		10
**Minimum temperature of the coldest month (°C)**	3.8		7.7
**Maximum temperature in February (°C)**	2.9		0.2
**Annual temperature range (°C)**	2.7		5
**Maximum temperature in December (°C)**	2.6		1.1
**Annual precipitation (mm)**	2.3		2.7
**Maximum temperature in September (°C)**	1.7		0.4
**Altitude (m)**	1.3		2.2
**Maximum temperature of the warmest month (°C)**	1.2		2.7
**Mean temperature of the driest quarter (°C)**	1.1		7.5
**Mean temperature in May (°C)**	1.1		21.8
**Minimum temperature in November (°C)**	0.8		0.6
**Minimum temperature in April (°C)**	0.7		0.3
**Precipitation of the driest month (mm)**	0.6		1.7
**Precipitation in December (mm)**	0.6		7.4
**Minimum temperature in March (°C)**	0.4		1.7
**Maximum temperature in November (°C)**	0.1		0.1

### Predicting the distribution of Psa in China

**Fig 4:**
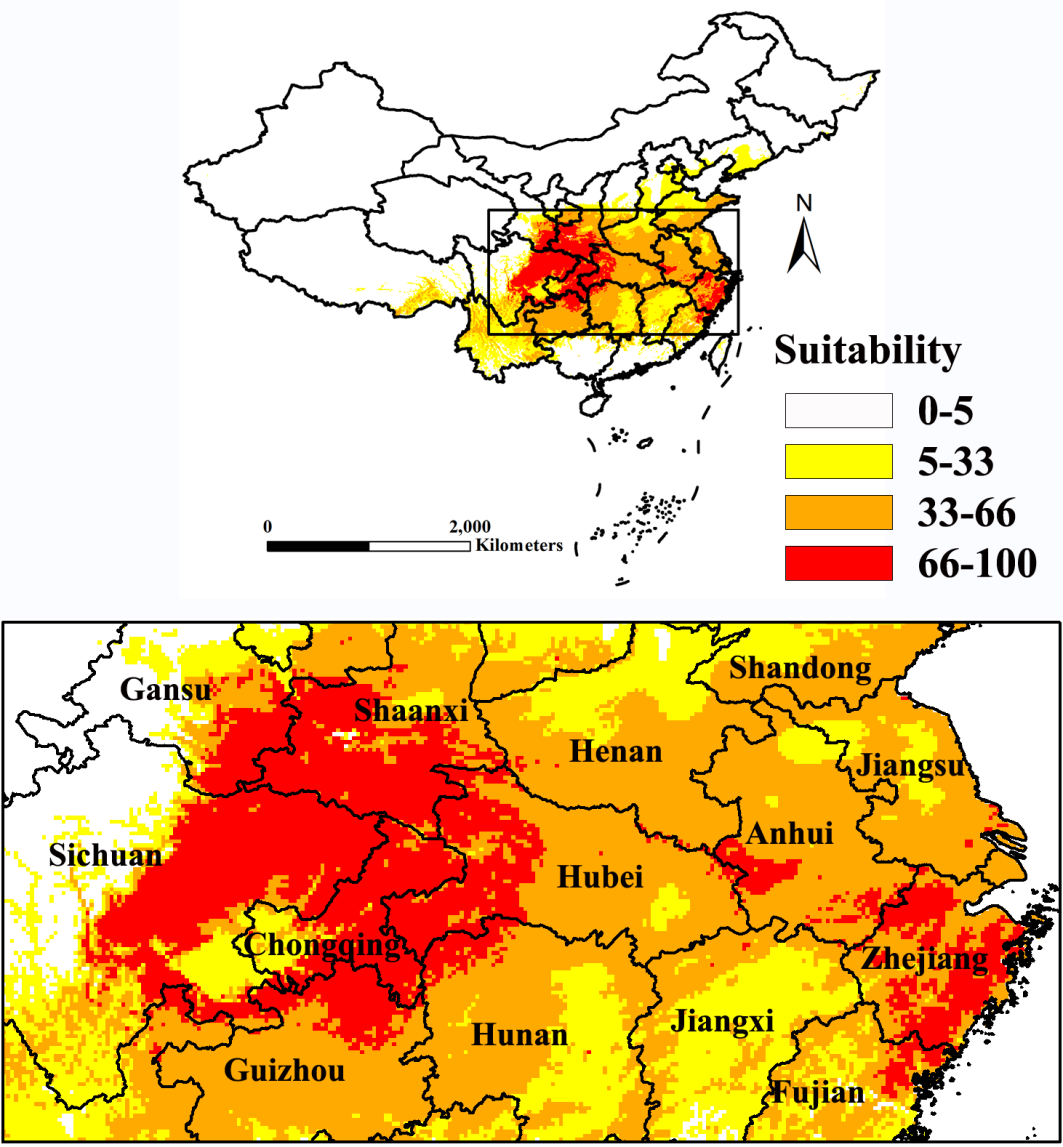
Distribution of core suitable areas of Psa under current climate condition in China. The probability of Psa is shown in the color scale in the legend. Red indicates highly suitable area with >66 probability of occurrence, orange indicates moderately suitable area with 33–66 probability of occurrence, yellow indicates poorly suitable area with 5–33 probability of occurrence and white indicates unsuitable area.

ArcGIS 10.0 was used to analyze the simulation results from the MaxEnt model for further study. The result showed that the area from latitude 25° to 36°N and from longitude 101° to 122°E was the primary potential suitable region of Psa in China. Based on the division criteria of suitability for Psa, the main suitable regions of Psa in China were extracted by ArcGIS (Fig 4). The potential suitable areas were mainly located in the provinces of Sichuan, Shaanxi, Chongqing, Hubei, Zhejiang, Gansu, Guizhou, Hunan, Jiangsu, Henan and Anhui, which occupied 27.78% of the land of China. The highly suitable area (with suitability values between 66 and 100) was mainly concentrated in Northeast Sichuan, South Shaanxi, most of Chongqing, West Hubei and Southwest Gansu. The areas of highly suitable areas in the major producing provinces were analyzed statistically (Table 2), and it showed that the most suitable areas of Psa occupied 4.94% of the land of China. Sichuan (1.38%), Shaanxi (0.84%), Hubei (0.6%), Chongqing (0.59%) and Zhejiang (0.48%) were considered to be the major suitable provinces for Psa.

**Table 2:** Analysis of highly suitable main distributions of Psa.

Province	Highly suitable (km^2^)	Total (km^2^)	Percentage (of highly suitable areas in the province, %)	Percentage (of highly suitable areas in China, %)
**Sichuan**	133055.6	455139	29.23	1.38
**Shaanxi**	81111.11	204167	39.73	0.84
**Chongqing**	56319.44	77083	73.06	0.59
**Hubei**	57569.44	175556	32.79	0.60
**Zhejiang**	46111.11	94816	48.63	0.48
**Gansu**	29166.67	414930	7.03	0.30
**Guizhou**	28680.56	159722	17.96	0.30
**Anhui**	16319.44	133680	12.21	0.17
**Hunan**	12083.33	194374	6.22	0.13
**Henan**	2013.89	161180	1.25	0.02
**China**	475486.11	9618680	—	4.94

### Area change, shift in distance and direction of mean centers of Psa under climate change scenarios

**Fig 5:**
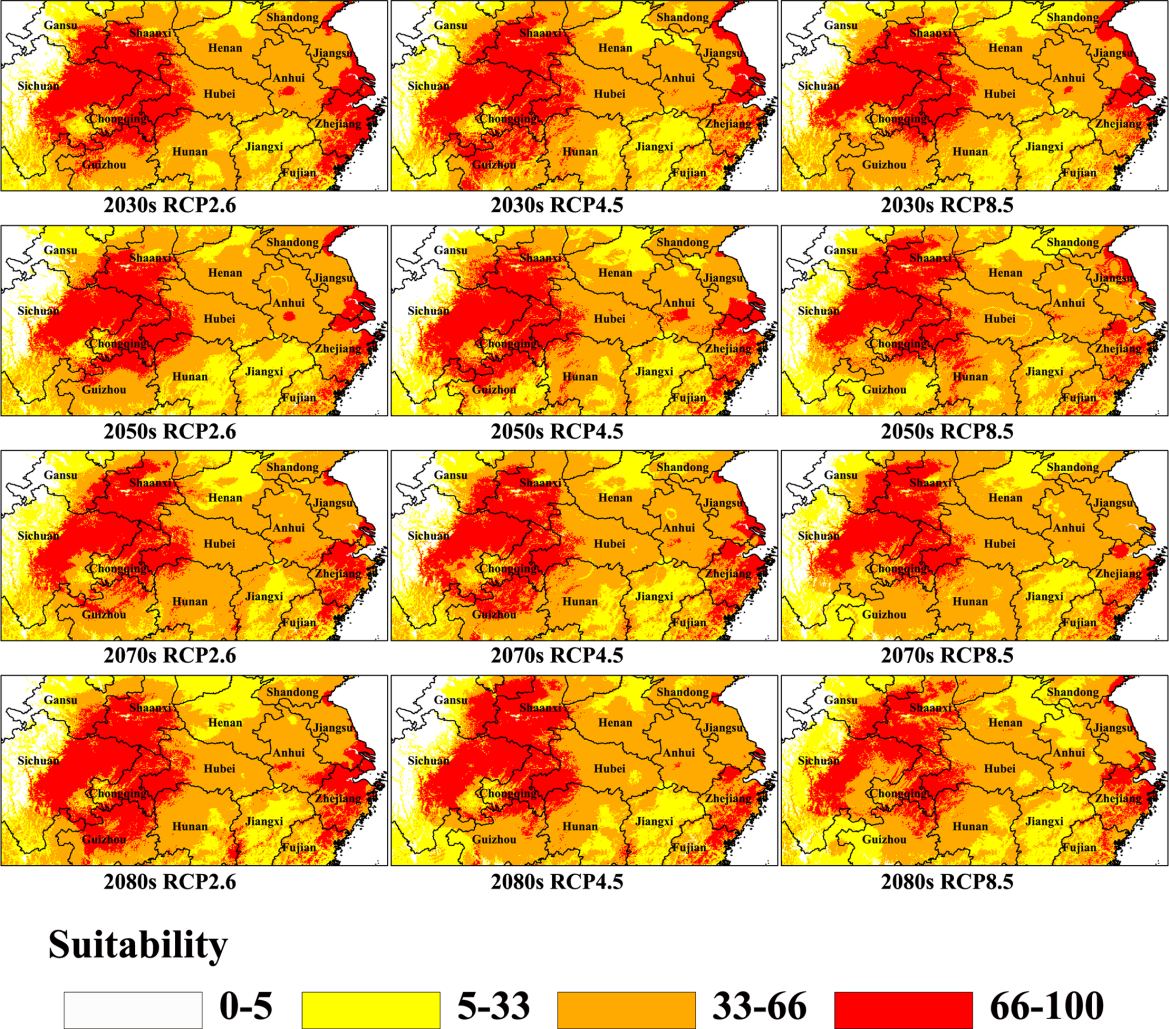
Distribution of core suitable areas of Psa under different climate change scenarios in China. The probability of Psa is shown in the color scale in the legend. Red indicates highly suitable area with >66 probability of occurrence, orange indicates moderately suitable area with 33–66 probability of occurrence, yellow indicates poorly suitable area with 5–33 probability of occurrence and white indicates unsuitable area of occurrence.

**Fig 6:**
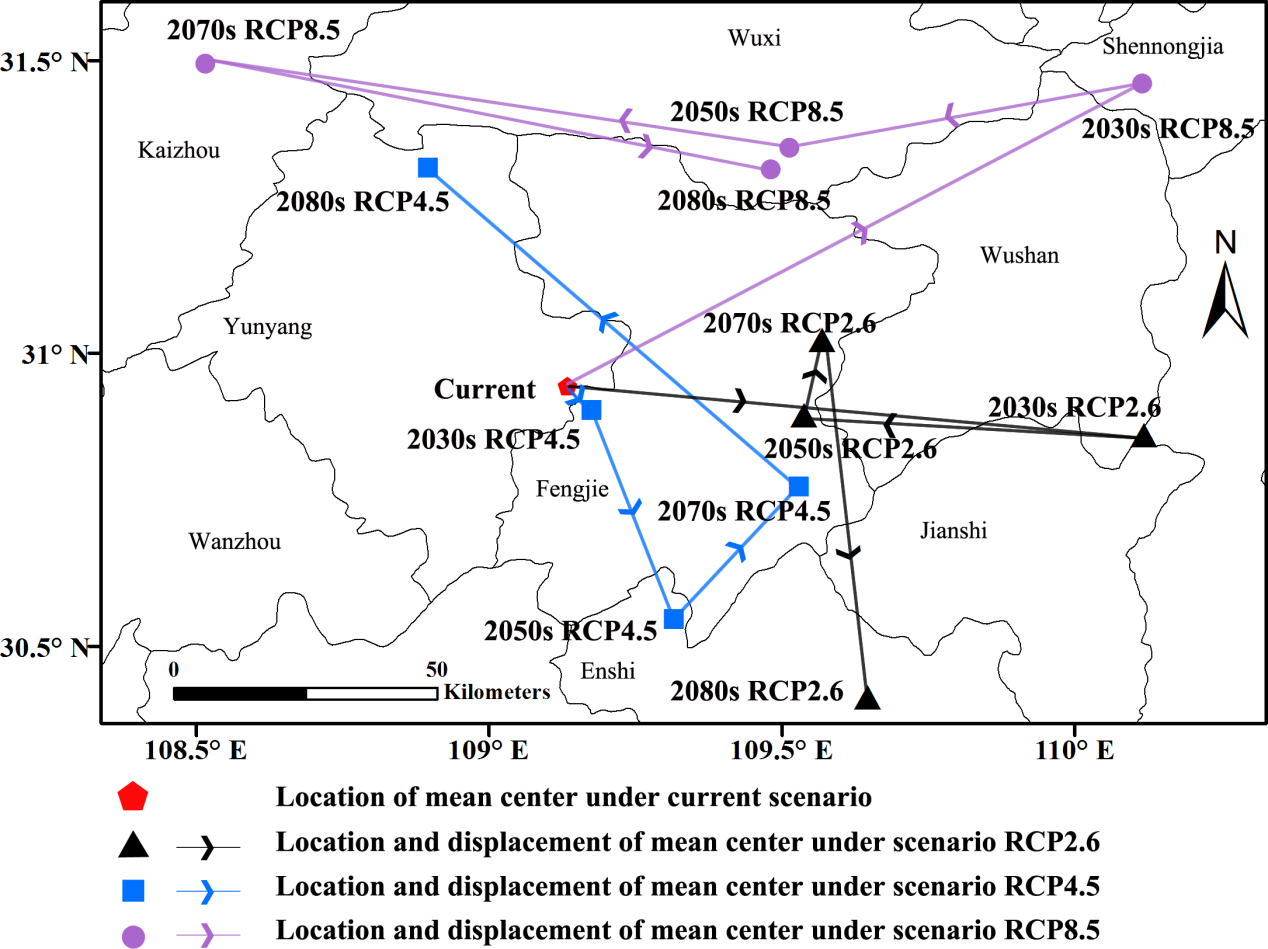
Center displacement of highly suitable areas during different periods.

Under scenario RCP2.6 (Table 3 and Fig 5), comparing the future suitable areas with the current suitable areas showed that areas of high suitability would have the greatest increase in the 2080s; the increase would be 11.71×10^4^ km^2^ and account for 124.63% of the current predicted area. From the present to the 2080s, the mean centers of highly suitable areas would shift from Yunyang (current) to Jianshi (2030s), Fengjie (2050s and 2070s) and Enshi (2080s). The shift in distance of the mean centers from the present location to the simulated location in the 2080s is approximately 73.87 km to the southeast (Table 4 and Fig 6).

**Table 3:** Predicted suitable areas for Psa under current and future climatic conditions.

Decade	Scenarios	Predicted area/×10^4^ km^2^			Account of the proportion of current predicted area (%)		
		Poorly	Moderately	Highly	Poorly	Moderately	Highly
**Current**	—	102.17	118.13	47.55	—	—	—
**2030s**	RCP2.6	102	119.41	56.89	99.83	101.09	119.64
	RCP4.5	114.64	115.55	54.57	112.2	97.81	114.77
	RCP8.5	111.22	118.01	47.51	108.85	99.89	99.92
**2050s**	RCP2.6	114.74	124.18	48.41	112.3	105.11	101.81
	RCP4.5	106.69	111.06	52.28	104.42	94.01	109.96
	RCP8.5	123.56	117.95	46.37	120.94	99.84	97.52
**2070s**	RCP2.6	120.95	130.33	47.95	118.38	110.32	100.85
	RCP4.5	115.83	126.8	49.96	113.37	107.34	105.06
	RCP8.5	129.68	131.97	37.89	126.92	111.72	79.71
**2080s**	RCP2.6	112.06	119.01	59.26	109.68	100.71	124.63
	RCP4.5	104.47	124.57	51.03	102.24	105.54	107.32
	RCP8.5	166.13	123.47	44.1	162.6	104.52	92.74

**Table 4:** Shifts in distance and direction of the mean centers of highly suitable areas in different periods.

Period	RCP2.6		RCP4.5		RCP8.5	
	Displacement (km)	Direction	Displacement (km)	Direction	Displacement(km)	Direction
**From T1 to T2**	98.89	Southeast	5.84	Southeast	111	Northeast
**From T2 to T3**	58.22	Northwest	38.38	Southeast	61.26	Southwest
**From T3 to T4**	13.24	Northeast	31.05	Northeast	100.83	Northwest
**From T4 to T5**	61.53	Southeast	83.58	Northwest	98.31	Southeast
**From T1 to T5**	73.87	Southeast	44.31	Northwest	50.83	Northeast

Scenario RCP4.5 indicated that the highly suitable areas would increase 7.02×10^4^ km^2^, 4.73×10^4^ km^2^, 2.41×10^4^ km^2^, 3.48×10^4^ km^2^, respectively (Table 3 and Fig 5). The mean centers would shift from Yunyang (current) to Fengjie (2030s), Enshi (2050s), Fengjie (2070s) and Yunyang (2080s). The shift in distance of the mean centers from the present location to the simulated location in the 2080s is approximately 44.31 km to the northeast (Table 4 and Fig 6).

Under scenario RCP8.5 (Table 3 and Fig 5), the highly suitable areas showed a decreasing trend from the present to the 2080s, and the reductions would be 0.04×10^4^ km^2^, 1.18×10^4^ km^2^, 9.66×10^4^ km^2^ and 3.45×10^4^ km^2^, respectively. The mean centers of highly suitable areas would shift from Yunyang (Current) to Shengnongjia (2030s), Wuxi (2050s), Kaizhou (2070s) and Wuxi (2080s). The shift in distance of the mean centers from the present to the 2080s is approximately 50.83 km to the northeast (Table 4 and Fig 6).

### Response of variables to suitability

**Fig 7:**
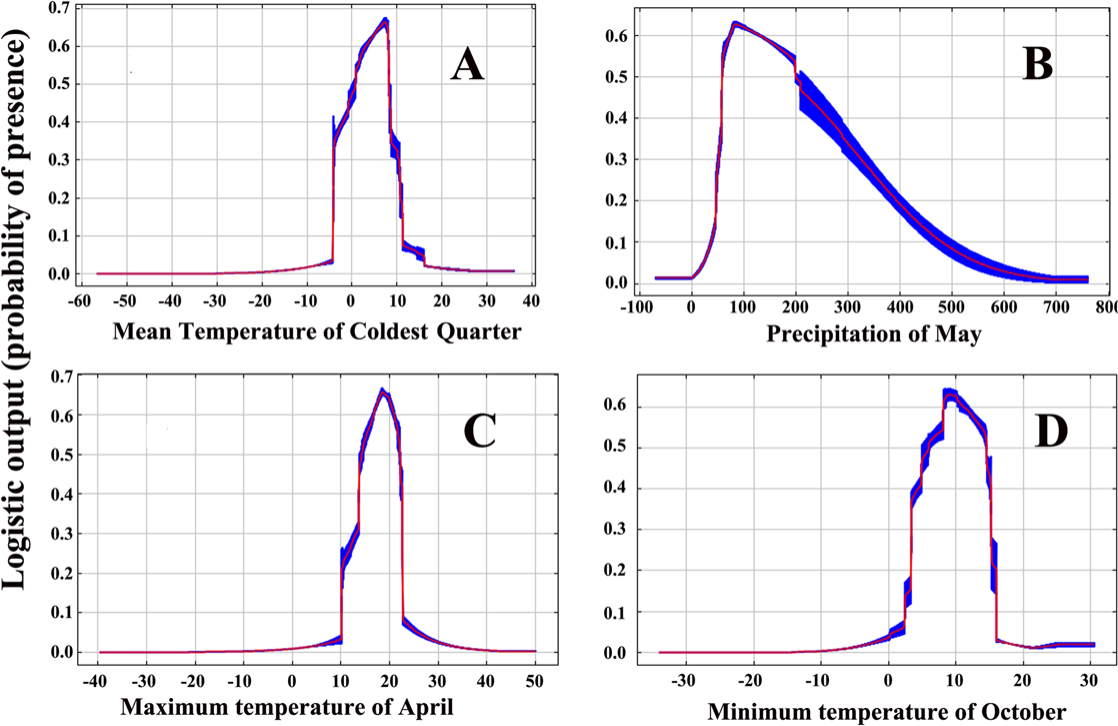
Response curves of the variables contributing most to the prediction by the MaxEnt models for Psa. (A) Mean Temperature of Coldest Quarter (bio11; °C). (B) Precipitation of May (prec5; mm), (C) Maximum temperature of April (tmax4; °C), (D) Minimum temperature of October (tmin10; °C).

Response curves indicated the relationships between environmental variables and the predicted probability of the presence of Psa. Individual response curves for different variables (model created using only the corresponding variable) showed that the predicted probability of the presence of Psa showed a similar pattern to the Poisson distribution (Table 5 and Fig 7).

**Table 5:** The suitable range of dominant environmental variables affecting the potential distribution of Psa.

Environmental variables	Suitable range	Optimum value
**Maximum temperature in April (°C)**	13.7–23.6	21.2
**Mean Temperature of the Coldest Quarter (°C)**	-3.9–9.3	8.1
**Precipitation in May (mm)**	50.1–317	83
**Minimum temperature in October (°C)**	3.6–17.1	10.3

According to the response curve of mean temperature of the coldest quarter, the probability of Psa occurrence increased up to 8.1°C and deceased sharply after that. Similar trends were observed for maximum temperature in April, and the response curve indicated that Psa would survive in locations where the maximum temperature in April was between 13.7–23.6°C; however, the probability of Psa decreased rapidly above 21.2°C. The response curve of precipitation in May indicated that higher levels of precipitation (50.1–317 mm) in May would be conducive for the development of cankers caused by Psa. The response curve of the minimum temperature in October showed that Psa can tolerate a wider range of temperatures (3.6 to 17.1°C) in October.

## Discussion

### Model selection and evaluation

At present, most studies researching Psa are concentrated on small-scale ranges, while there are relatively few studies that examine the potential geographic distribution of Psa on large scales and model future niches under climate change scenarios. Based on the maximum entropy principle, the MaxEnt software uses the species distribution data and the environmental variables to analyze the distribution state of the species when the entropy is the largest [76]. Numerous studies show that MaxEnt performs better than other niche models and has advantages, such as short running time, easy operation, small sample size and high simulation precision [68, 77–79]; therefore, this study was designed to examine the large scale and future distribution of Psa using MaxEnt theory.

The results showed that the choice of environmental variables has a certain influence on the prediction results of niche models. Many researchers that use the MaxEnt model to predict the geographical distribution of species non-selectively use all of the environmental factors or the major environmental factors [59, 80–82]. The environmental variables, which were obtained from the WorldClim database and CCAFS, are based on temperature and rainfall data based on the different needs of the occurrence calculations. Therefore, there are inevitable relationships between the auto correlation of these variables, multiple linear repetition and other issues. Studies have confirmed that these highly relevant variables introduce redundant information into the model prediction process, which affects the prediction results [65, 83, 84]. To avoid these problems when modeling, relevant analysis and effective screening of environmental variables should be carried out before subsequent analyses occurs. In this study, the importance of the variables was evaluated by examining the contribution rate of each factor to species distribution. The environmental variables with small contribution rates were excluded, and the correlation of the selected variables was analyzed using only the leading the environmental variables; additionally, the model was reconstructed to reduce the impact of redundant information on the simulation results and to improve the accuracy of the prediction results.

At present, the most widely used method for model accuracy evaluation is the ROC curve method (AUC method). Because AUC is not affected by diagnostic thresholds, as it provides performance evaluation results at all threshold ranges, it is now recognized as a niche model evaluator. AUC values range from 0.5 to 1, where the closer the value is to 1, the higher the accuracy of the model [85, 86]. In this study, the AUC average values of the simulated training set based on the dominant environmental variables were greater than 0.949 (i.e., very close to 1), and the predicted results reached the "excellent" level, indicating that the geographical distribution of the predicted model is in high agreement with the actual distribution. In addition, this study used ArcGIS to postulate the raster files of the MaxEnt output so that the distribution data of the target species and the environmental variable data corresponded to the grid cells, effectively reduced system error, and further improved the accuracy of the data.

### Predicting the distribution of Psa in China

The present use of GIS technology to simulate the spatial distribution patterns of species is an important tool. More and more studies have used GIS and statistical analysis methods to identify the relationship between species richness and spatial heterogeneity [87–89]. In this study, a combination of MaxEnt and ArcGIS was used to predict the potential geographic distribution of Psa in China. The results showed that, under current climatic conditions, the area from latitude 25° to 36°N and from longitude 101° to 122°E is the primary potential suitable area of Psa in China. The highly suitable area was mainly concentrated in Northeast Sichuan, South Shaanxi, most of Chongqing, West Hubei and Southwest Gansu and occupied 4.94% of land in China. From 2014 to 2016, we investigated the occurrence of kiwifruit bacterial canker in Sichuan and Shaanxi provinces. The result showed that the disease is mainly distributed in Guangyuan, Bazhong, Mianyang, Chengdu, Yaan and Yibin in Sichuan province, and in Xian, Baoji and Weinan in Shaanxi province. The potential distribution of this study was highly coincident with the locations of field surveys in Shaanxi and Sichuan. In an earlier study, Shao *et al* [19] simulated the potential distribution of Psa at the state level using a fuzzy mathematics comprehensive evaluation. The results showed that potential areas of the pathogen were mainly distributed in Sichuan, Yunnan, Guizho, Fujian, Anhui, Hunan, Hubei, Henan, Jiangxi, Shaanxi, Zhejiang, Chongqing and Tibet. Our model predictions are aligned with the predictions of Shao et al [19].in most of the kiwifruit growing areas, but they significantly differed in other areas such as Yunnan and Tibet. The differences could be due to the model simulation theory and specific assumptions, types of environmental variables and calibration settings. For instance, as a type of correlative model, fuzzy mathematics comprehensive evaluation based on direct measures of physiological variables ignores biotic interactions, while MaxEnt is based on observations and includes the effects of biotic interactions.

In this study, the area under three climate change scenarios was statistically analyzed with ArcGIS to identify the trends of the area impacted by disease. The results showed that under both scenario RCP2.6 and scenario RCP4.5, suitable areas of Psa would increase until the 2080s; in contrast, under scenario RCP8.5, the highly suitable areas decreased from the present until the 2080s. This indicated that different emission scenarios have different and opposite effects on the potential distribution of Psa in China.

Climate is a decisive factor in species distribution, while changes in species distribution patterns are the most clear and direct reflection of climate change. Climate change characteristics influenced by global warming have been changing the structure and function of terrestrial ecosystems, thus changing the biological habitats and geographical distribution of species [32, 36, 38, 41]. To understand the response of Psa to climate change, we calculated and analyzed the position of the mean center in different grades and the center’s shift over time based on the calculation method used by Yue et al [75]. The results showed that mean centers of highly suitable areas will change in a variety of ways under the three emission scenarios until the 2080s. Under scenario RCP4.5 and scenario RCP8.5, the mean centers will move to the northwest. The movement may be related to the increase in the average temperature and precipitation. In addition, from the simulation results, no obvious regularities were found in the location and displacement of the mean centers under the different scenarios. This may be due to the lack of continuity between current and future climate variables. The current period is from 1950 to 2000, while the future period is from 2030 to 2080, and there is a gap between 2000 and 2030. The reciprocating movement of the mean center may be due to the lack of data during this period.

### Effects of climatic factors on the distribution of Psa

Studying the interaction between species and the environment is an essential aspect of species ecology[90]. The relationship between the probability of species presence and dominant environmental variables was analyzed in this paper, and the response curves were created by MaxEnt. The analysis showed that the probability of species presence changed as a result of the dominant environmental variables (maximum temperature in April, mean temperature of the coldest quarter, precipitation in May and minimum temperature in October). Both the precipitation and temperature variables were strongly correlated with the distribution of Psa. Our results were in good agreement with some previous research conclusions. For example, previous research by Marcelletti and Scortichini found low temperature, abundant rainfall and high humidity were the most favorable conditions for disease development [91], and this is in accordance with the trends we observed in the response curve of precipitation in May. The model results showed that the probability that Psa would be present decreased rapidly above 21.2°C, which is consistent with the previous experimental findings of Serizawa and Ichikawa, who found that once temperatures exceeded 25°C, the harm caused by Psa weakened [91]. Other research showed that when the average temperature reached 20°C, the spread of the disease was inhibited [92]. Overall, these studies suggested that the occurrence of Psa is closely related to climate, and further studies about this will be useful for predicting and forecasting the kiwifruit canker.

### Limitations in this research and the future directions

Although the MaxEnt model predicts the advantages of simple operation, small sample demand and high prediction accuracy, there are some limitations that are similar to other niche prediction models.

1. The environmental variables used in the prediction by the MaxEnt model are all climatic variables except for altitude. The 19 bioclimatic variables are the climatic extremes, i.e., the maximum and the minimum of the actual distribution of Psa. The MaxEnt model shows the maximum likelihood of species distribution and cannot be prepared to express species in the main areas of actual distribution. The above forecast results are more focused on understanding and demonstrating the potential geographical distribution of Psa and revealing the climate characteristics suitable for the distribution of species.
2. The basic niche is an ideal niche, which refers to the maximum niche that a species occupies under ideal living conditions without competition by any other species. The theory only needs to consider the influence of abiotic factors. When the prediction of the suitable area is based not only on the demand of the species in the niche but also on the actual living environment, the biological factors (such as the interaction between species, the vegetation type, geomorphological features, the species own diffusion ability and the soil type) will also have a significant impact on the potential distribution of the predicted species. Based on the above reasons, it can be deduced that the model predicts a niche that is larger than the actual niche occupied by Psa. In this regard, the next step, in addition to considering the impact of climate factors, should consider the interaction between species and other biological factors expressed in order to improve the model’s predictive effect.
3. Over the past 30 years, with the continuous discharge and maintenance of greenhouse gases, the global climate is warming abnormally, and climate change can cause changes in species growth and distribution patterns. The environmental variables used in this study were derived from the world climate database, the WorldClim, which includes data from 1950-2000s; however, this database is missing recent climate data for at least the past 10 years. In the future, the missing data should be filled in so that the forecast results are more accurate and reliable.

## Conclusions

In this study, we successfully modeled the current niches of Psa in China, as well as future niches under three climate change scenarios, which allowed for the identification of climatic variables important for the potential establishment of Psa. This study concludes that under scenario RCP2.6 and RCP4.5, the habitat suitability of Psa will increase until the 2080s. We suggest that future climate scenarios should be included in the control measures of Psa, which were created by the institutions responsible for agricultural management.

## Supporting information

S1 Table List of locations used for this study, with longtitude, latitude and sources. (DOCX)

S2 Table List of environmental variables used for this study, with type and measurement unit. (DOCX)

S3 Table The evaluation criterion of AUC. (DOCX)
