# Supplementary material for: Modeling and mapping the current and future distribution of Pseudomonas syringae pv. actinidiae under climate change in China
Source: PLoS One. 2018 Feb 1;13(2):e0192153. doi: 10.1371/journal.pone.0192153 (PMC5794145; doi:10.1371/journal.pone.0192153)
Supplement: S1 Table — (DOCX) [file pone.0192153.s001.docx]

**S1 Table. List of locations used for this study, with longtitude, latitude and sources**

| **County** | **Location**  **(Province or Region)** | **Longitude** | | **Latitude** | **sources** |  |
| --- | --- | --- | --- | --- | --- | --- |
| China | Yaan, Sichuan | | 102.5 | 30.5 | EPPO |  |
| China | Chengdu, Sichuan | | 103.59 | 30.95 | the auhtor surveys |  |
| China | Chengdu, Sichuan | | 103.717 | 31.026 | the auhtor surveys |  |
| China | Guangyuan, Sichuan | | 105.447 | 32.11 | the auhtor surveys |  |
| China | Guangyuan, Sichuan | | 105.886 | 32.107 | the auhtor surveys |  |
| China | Guangyuan, Sichuan | | 106.2503 | 31.9858 | the auhtor surveys |  |
| China | Guangyuan, Sichuan | | 105.467 | 31.98 | the auhtor surveys |  |
| China | Guangyuan, Sichuan | | 105.933 | 31.95 | the auhtor surveys |  |
| China | Yibin, Sichuan | | 105.1483 | 28.124 | the auhtor surveys |  |
| China | Mianyang, Sichuan | | 104.55 | 31.774 | the auhtor surveys |  |
| China | Mianyang, Sichuan | | 104.38 | 30.99 | the auhtor surveys |  |
| China | Yaan, Sichuan | | 103.044 | 30.126 | the auhtor surveys |  |
| China | BaZhong, Sichuan | | 106.597 | 31.752 | the auhtor surveys |  |
| China | BaZhong, Sichuan | | 107.147 | 31.927 | the auhtor surveys |  |
| China | BaZhong, Sichuan | | 107.465 | 32.055 | the auhtor surveys |  |
| China | BaZhong, Sichuan | | 107.415 | 32.10868 | the auhtor surveys |  |
| China | Leshan, Sichuan | | 103.94621 | 28.8766 | the auhtor surveys |  |
| China | Yaan, Sichuan | | 103.3511 | 30.213 | the auhtor surveys |  |
| China | Yaan, Sichuan | | 102.8957 | 32.0205 | the auhtor surveys |  |
| China | Chengdu, Sichuan | | 103.7956 | 31.133144 | the auhtor surveys |  |
| China | Yibin, Sichuan | | 104.933 | 28.915 | the auhtor surveys |  |
| China | Baoji, Shaanxi | | 107.44 | 34.16 | the auhtor surveys |  |
| China | Xian, Shaanxi | | 108.05 | 34.16 | the auhtor surveys |  |
| China | Xian, Shaanxi | | 108.12 | 34.09 | the auhtor surveys |  |
| China | Xian, Shaanxi | | 108.82 | 34.12 | the auhtor surveys |  |
| China | Xian, Shaanxi | | 108.97 | 34.1 | the auhtor surveys |  |
| China | Baoji, Shaanxi | | 107.35 | 37.45 | the auhtor surveys |  |
| China | Baoji, Shaanxi | | 107.63 | 34.44 | the auhtor surveys |  |
| China | Baoji, Shaanxi | | 107.52 | 34.29 | the auhtor surveys |  |
| China | Yanan, Shaanxi | | 109 | 36 | EPPO/CABI |  |
| China | Xian, Shaanxi | | 109.01 | 34.03 | the auhtor surveys |  |
| China | Weinan, Shaanxi | | 109.57 | 34.45 | the auhtor surveys |  |
| China | Hanzhogn, Shaanxi | | 107.11 | 33.14 | the auhtor surveys |  |
| China | Hangzhou, Zhejiang | | 120.19 | 30.31 | [[1](#_ENREF_1)] |  |
| China | Jiangshan, Zhejiang | | 118.82 | 28.81 | [[2](#_ENREF_2)] |  |
| China | Wenzhou, Zhejiang | | 120.08 | 27.59 | [[2](#_ENREF_2)] |  |
| China | Ninghai, Zhejiang | | 121.5 | 29.36 | [[2](#_ENREF_2)] |  |
| China | Shangyu, Zhejiang | | 121.11 | 30.07 | [[2](#_ENREF_2)] |  |
| China | Tongxiang, Zhejiang | | 120.5 | 30.62 | [[2](#_ENREF_2)] |  |
| China | Suichang, Zhejiang | | 119.17 | 28.7 | [[2](#_ENREF_2)] |  |
| China | Xixia, Henan | | 111.27 | 33.65 | [[3](#_ENREF_3)] |  |
| China | Kaizhou, Chongqing | | 108.22 | 31.24 | [[3](#_ENREF_3)] |  |
| China | Wanzhou, Chongqing | | 108.41 | 30.97 | [[3](#_ENREF_3)] |  |
| China | Nanchuan, Chongqing | | 107.01 | 29.31 | [[3](#_ENREF_3)] |  |
| China | Changsha, Hunan | | 112 | 28 | EPPO |  |
| China | Changde, Hunan | | 110.7 | 29.92 | [[4](#_ENREF_4)] |  |
| China | Jianghua, Hunan | | 111.56 | 25.19 | [[5](#_ENREF_5)] |  |
| China | Changfeng, Ahui | | 117 | 32 | EPPO/CABI |  |
| China | AnQing, Anhui | | 116.46 | 31.02 | [[4](#_ENREF_4)] |  |
| China | Jiexiu, Shanxi | | 112 | 37 | EPPO |  |
| China | Xiuwen, Guizhou | | 106.48 | 26.93 | [[6](#_ENREF_6)] |  |
| China | Shuicheng, Guizhou | | 104.85 | 26.59 | [[6](#_ENREF_6)] |  |
| China | Jianhu, Jiangsu | | 119.93 | 33.45 | [[4](#_ENREF_4)] |  |
| China | Jianshi, Hubei | | 109.73 | 30.6 | [[6](#_ENREF_6)] |  |
| Japan | shizuoka | | 138.23 | 34.59 | [[7](#_ENREF_7)] |  |
| Japan | kanagawa | | 139.43 | 35.31 | [[7](#_ENREF_7)] |  |
| Japan | Wakayama | | 135.1 | 34.14 | [[7](#_ENREF_7)] |  |
| Japan | Chiba | | 140.07 | 35.36 | [[7](#_ENREF_7)] |  |
| Japan | Takikawa | | 141.56 | 43.36 | [[8](#_ENREF_8)] |  |
| Japan | Nakajima | | 139.35 | 35.51 | [[8](#_ENREF_8)] |  |
| Japan | Serizawa | | 139.25 | 35.22 | [[8](#_ENREF_8)] |  |
| Japan | Hokkaido | | 141.347 | 43.064 | EPPO |  |
| Japan | Chiyoda | | 139.75 | 35.68 | EPPO |  |
| Japan | Saga | | 130.18 | 33.14 | [[9](#_ENREF_9)] |  |
| Korea | Jeonnam | | 127 | 34.45 | [[10](#_ENREF_10)] |  |
| Korea | Wando | | 126.41 | 34.21 | [[10](#_ENREF_10)] |  |
| Korea | Haenam | | 126.31 | 34.32 | [[10](#_ENREF_10)] |  |
| Korea | Sunchon | | 126.38 | 35.11 | [[10](#_ENREF_10)] |  |
| Korea | Bukjeju | | 126.36 | 33.23 | [[10](#_ENREF_10)] |  |
| Korea | Goheung | | 127.15 | 34.36 | [[11](#_ENREF_11)] |  |
| Korea | Jangheung | | 126.56 | 34.42 | [[11](#_ENREF_11)] |  |
| Korea | Bosung | | 127.03 | 37.58 | [[11](#_ENREF_11)] |  |
| Korea | Muan | | 126.28 | 34.59 | [[11](#_ENREF_11)] |  |
| Korea | Suncheon | | 127.29 | 34.56 | [[11](#_ENREF_11)] |  |
| Korea | Gyeongnam | | 128.15 | 35.15 | [[11](#_ENREF_11)] |  |
| Korea | Seogwipo-si | | 126.29 | 33.17 | [[12](#_ENREF_12)] |  |
| Greece | Drosero | | 22.14 | 40.49 | [[13](#_ENREF_13)] |  |
| Slovenia | Primorska | | 13.54 | 45.72 | [[14](#_ENREF_14)] |  |
| Slovenia | Žalec | | 15.167 | 46.25 | EPPO |  |
| Slovenia | Dolina | | 14.3 | 45.39 | [[15](#_ENREF_15)] |  |
| Turkey | Avanos | | 35 | 39 | EPPO |  |
| Turkey | Rize | | 40.31 | 41.01 | [[16](#_ENREF_16)] |  |
| Switzerland | Geneva canton | | 6.08 | 46.12 | [[15](#_ENREF_15)] |  |
| Switzerland | Romoos | | 8.01427 | 47.0002 | EPPO |  |
| France | Aquitaine | | 1 | 44.35 | [[17](#_ENREF_17)] |  |
| France | Rhone Alpes | | 5.22 | 45.28 | [[17](#_ENREF_17)] |  |
| France | Pays de la Loire | | -0.33 | 47.28 | [[17](#_ENREF_17)] |  |
| France | Poitou-Charentes | | -0.04 | 46.1 | [[17](#_ENREF_17)] |  |
| France | Midi-Pyrénées | | 1.3 | 43.45 | [[17](#_ENREF_17)] |  |
| France | Centre-Val de Loire | | 1.45 | 47.3 | [[17](#_ENREF_17)] |  |
| France | Rhone Alpes | | 5.36 | 44.88 | [[15](#_ENREF_15)] |  |
| France | PACA | | 5.27 | 43.17 | [[18](#_ENREF_18)] |  |
| France | PACA | | 5.22 | 44.89 | [[18](#_ENREF_18)] |  |
| France | Limousin | | 1.42 | 45.46 | [[18](#_ENREF_18)] |  |
| France | Mayenne | | -0.37 | 48.18 | [[18](#_ENREF_18)] |  |
| France | Corsica | | 8.59 | 41.59 | [[15](#_ENREF_15)] |  |
| France | Creuse | | 2 | 46 | EPPO |  |
| Portugal | Vila Boa de Quires | | -8.12 | 41.11 | [[19](#_ENREF_19)] |  |
| Portugal | Santa Maria da Feira | | -8.3 | 40.57 | [[19](#_ENREF_19)] |  |
| Portugal | Valenca | | -8.38 | 42.01 | [[19](#_ENREF_19)] |  |
| Portugal | Lago, Braga | | -8.24 | 41.37 | [[19](#_ENREF_19)] |  |
| Portugal | Entre-Douro-e-Mino | | -8.39 | 41.08 | [[15](#_ENREF_15)] |  |
| Portugal | Vale de abelha | | -8 | 39.5 | EPPO |  |
| Italy | Veneto, Treviso | | 11.45 | 45.3 | [[20](#_ENREF_20)] |  |
| Italy | Calabria | | 15.39 | 38.06 | [[21](#_ENREF_21)] |  |
| Italy | Emilia Romagna, Ravenna | | 10.59 | 44.32 | [[20](#_ENREF_20)] |  |
| Italy | Latina, Latium | | 12.54 | 41.27 | [[22](#_ENREF_22)] |  |
| Italy | Viterbo, Latium | | 12.06 | 42.25 | [[23](#_ENREF_23)] |  |
| Italy | Roma, Latium | | 12.3 | 41.53 | [[23](#_ENREF_23)] |  |
| Italy | Cuneo, Piedmont | | 7.32 | 44.23 | [[23](#_ENREF_23)] |  |
| Italy | Vercelli, Piedmont | | 8.25 | 45.19 | [[23](#_ENREF_23)] |  |
| Italy | Revello, Piedmont | | 7.23 | 44.39 | [[24](#_ENREF_24)] |  |
| Italy | Campiglione Fenile | | 7.19 | 44.48 | [[24](#_ENREF_24)] |  |
| Italy | Envie, Piedmont | | 7.22 | 44.4 | [[24](#_ENREF_24)] |  |
| Italy | Asti, Piedmont | | 8.12 | 44.54 | [[23](#_ENREF_23)] |  |
| Italy | Bagnolo | | 7.18 | 44.45 | [[24](#_ENREF_24)] |  |
| Italy | Andora | | 8.15 | 43.98 | [[18](#_ENREF_18)] |  |
| Italy | Friuli-Venezia Giula | | 13 | 46 | [[15](#_ENREF_15)] |  |
| Italy | Campania | | 14.12 | 40.55 | [[15](#_ENREF_15)] |  |
| Italy | Campello sul Clitunno | | \| 12.8333 \| 12.8333 \| \| --- \| --- \| | 42.8333 | EPPO/GBIF |  |
| Spain | Pontevedra | | -8.38 | 42.25 | [[25](#_ENREF_25)] |  |
| Spain | Galicia | | -7.51 | 42.45 | [[26](#_ENREF_26)] |  |
| Spain | Galicia | | -8.11 | 41.22 | [[15](#_ENREF_15)] |  |
| Spain | Yunclillos | | -4 | 40 | EPPO |  |
| Germany | Bavaria | | 11.34 | 48.08 | [[15](#_ENREF_15)] |  |
| Germany | Schleswig-Holstein | | 9.45 | 54.12 | [[15](#_ENREF_15)] |  |
| Georgia | Lanchkhuti | | 43.5 | 42 | EPPO |  |
| New Zealand | Pongakawa, Bay of plenty | | 176.28 | -37.5 | [[27](#_ENREF_27)] |  |
| New Zealand | Pukehina, Bay of plenty | | 176.31 | -37.49 | [[27](#_ENREF_27)] |  |
| New Zealand | Te Puke, Bay of plenty | | 176.19 | -37.46 | [[28](#_ENREF_28)] |  |
| New Zealand | Te Puke, Bay of plenty | | 176.35 | -37.56 | [[29](#_ENREF_29)] |  |
| New Zealand | Rangiuru, Bay of plenty | | 176.22 | -37.46 | [[27](#_ENREF_27)] |  |
| New Zealand | Katikati, Bay of plenty | | 175.55 | -37.33 | [[27](#_ENREF_27)] |  |
| New Zealand | Katikati, Bay of plenty | | 175.65 | -38.21 | [[29](#_ENREF_29)] |  |
| New Zealand | Te kaha, Bay of plenty | | 177.4 | -37.44 | [[29](#_ENREF_29)] |  |
| New Zealand | Te kaha, Bay of plenty | | 177.36 | -37.48 | [[28](#_ENREF_28)] |  |
| New Zealand | Tauranga, Bay of plenty | | 176.1 | -37.41 | [[27](#_ENREF_27)] |  |
| New Zealand | Christchurch-Canterbury | | 174 | -42 | EPPO/GBIF |  |
| Chile | Maule | | -71 | -30 | EPPO/GBIF |  |
| Chile | Maule | | -72.01 | -31.21 | [[27](#_ENREF_27)] |  |

Note: Samples of the auhtor surveys were tested by 16S-23S ribosomal DNA identification in our laboratory[[30](#_ENREF_30), [31](#_ENREF_31)].

1. Shi ZY, Zhang HQ, Xiao JP, Yang LQ, Sun ZW, Xie M, et al. The resistance evaluation of different kiwifruit varieties to canker. acta Agriculturae Zhejiangensis. 2014; 3: 752-759.

2. Zhang HQ, Li HM, Feng JJ, Xiao JP, Song GH, Xie M. Investigation and analysis of infection caused by Pseudomonas syringae pv. Actinidiae and its affecting factors in Zhejiang province. acta Agriculturae Zhejiangensis. 2013; 4: 832-835.

3. Zhou DX, Yin YP, Wang ZG, Xiong S. Establishment of a method to rapidly detect only viable cells of Pseudomonas syringae pv. actinidiae by EMAqPCR. Plant Protection. 2017; 3: 143-148.

4. Li Y, Cheng HY, Fang SM, Qian ZH. Prevalent forecast of kiwifruit bacterial canker caused by Pseudomonas syringae pv actinidiae. Chinese Journal of Applied Ecology. 2001; 3: 355-358. doi: 10.13287/j.1001-9332.2001.0084

5. Fang Y, Zhu X, Wang Y. Preliminary studies on kiwifruit diseases in Hunan Province. Sichuan Fruit Science Technology. 1990; 18: 28-29.

6. Li YZ, Song XB, Zhang XW. Sutdies on laws of occurrence of bacterial canker in kwifruit. Journal of Northwest Forestry University. 2000; 2: 53- 56.

7. Serizawa S, Ichikawa T, Takikawa Y, Tsuyumu S, Goto M. Occurrence of bacterial canker of kiwifruit in Japan: description of symptoms, isolation of the pathogen and screening of bactericides. Japanese Journal of Phytopathology. 1989; 4: 427-436. doi: 10.3186/jjphytopath.55.427

8. Koh YJ, Nou IS. DNA markers for identification of Pseudomonas syringae pv. actinidiae. Molecules & Cells. 2002; 2: 309-314. PMID: 12018854

9. Fujikawa T, Sawada H. Genome analysis of the kiwifruit canker pathogen Pseudomonas syringae pv. actinidiae biovar 5. Sci Rep. 2016; : 21399. doi: 10.1038/srep21399

10. Han HS, Koh YJ, Hur JW, Jung JS. Identification and Characterization of Coronatine-Producing Pseudomonas syringae pv. actinidiae. Journal of Microbiology & Biotechnologygy. 2003; 11: 6423-6430.

11. Ko SJ, Lee YH, Cha KH, Lee SD, Kim KC. Occurrence of Kiwifruit Bacterial Canker Disease and Control by Cultivation Ope. Plant Pathology Journal. 2002; 3: 179-183. doi: 10.5423/RPD.2002.8.3.179

12. Koh YJ, Kim GH, Jung JS, Lee YS, Hur JS. Outbreak of bacterial canker on Hort16A (Actinidia chinensis Planchon) caused by Pseudomonas syringae pv. actinidiae in Korea. New Zealand Journal of Crop & Horticultural Science. 2010; 4: 275-282.

13. Holeva MC, Glynos PE, Karafla CD. First report of bacterial canker of kiwifruit caused by Pseudomonas syringae pv. actinidiae in Greece. Plant Disease. 2015; 5: 150113075012005. doi: 10.1094/PDIS-07-14-0738-PDN

14. Dreo T, Pirc M, Ravnikar M, ŽEžLina I, Poliakoff F, Rivoal C, et al. First report of Pseudomonas syringae pv. actinidiae, the causal agent of bacterial canker of kiwifruit in Slovenia. Plant Disease. 2014; 11: 1578. doi: 10.1094/PDIS-05-14-0513-PDN

15. Wilstermann A, Schrader G, Kehlenbeck H, Robinet C. Potential spread of kiwifruit bacterial canker (Pseudomonas syringae pv. actinidiae) in Europe. Eppo Bulletin. 2017; 2: 255-262. doi: 10.1111/epp.12385

16. Bastas KK, Karakaya A. First report of bacterial canker of kiwifruit caused by Pseudomonas syringae pv. actinidiae in Turkey. Plant Disease. 2012; 3: 452-452. doi: 10.1094/PDIS-08-11-0675

17. Cunty A, Poliakoff F, Rivoal C, Cesbron S, Fischer‐Le Saux M, Lemaire C, et al. Characterization of Pseudomonas syringae pv. actinidiae (Psa) isolated from France and assignment of Psa biovar 4 to a de novo pathovar: Pseudomonas syringae pv. actinidifoliorum pv. nov. Plant Pathology. 2015; 3: 582-596. doi: 10.1111/ppa.12297

18. Cunty A, Cesbron S, Poliakoff F, Jacques MA, Manceau C. Origin of the Outbreak in France of Pseudomonas syringae pv. actinidiae Biovar 3, the Causal Agent of Bacterial Canker of Kiwifruit, Revealed by a Multilocus Variable-Number Tandem-Repeat Analysis. Applied & Environmental Microbiology. 2015; 19: 6773-6789. doi: 10.1128/AEM.01688-15

19. Renzi M, Mazzaglia A, Balestra GM. Widespread distribution of kiwifruit bacterial canker caused by the European Pseudomonas syringae pv. actinidiae genotype in the main production areas of Portugal. Phytopathologia Mediterranea. 2012; 2: 402-409. doi: 10.14601/Phytopathol_Mediterr-9488

20. Biondi E, Galeone A, Kuzmanovi N, #x, Ardizzi S, Lucchese C, et al. Pseudomonas syringae pv. actinidiae detection in kiwifruit plant tissue and bleeding sap. Annals of Applied Biology. 2013; 1: 60–70. doi: 10.1111/aab.12001

21. Ferrante P, Scortichini M. Molecular and phenotypic variability among Pseudomonas avellanae, P. syringae pv. actinidiae and P. syringae pv. theae: the genomospecies 8 sensu gardan et al. (1999). Journal of Plant Pathology. 2011; 3: 659-666.

22. Scortichini M. Occurrence of Pseudomonas syringae pv. actinidiae on kiwifruit in Italy. Plant Pathology. 2010; 6: 1035-1038. doi: 10.1111/j.1365-3059.1994.tb01654.x

23. Marcelletti S, Scortichini M. CLONAL OUTBREAKS OF BACTERIAL CANKER CAUSED BY PSEUDOMONAS SYRINGAE pv. ACTINIDIAE ON ACTINIDIA CHINENSIS AND A. DELICIOSA IN ITALY. Journal of Plant Pathology. 2011; 2: 479-483.

24. Prencipe S, Nari L, Vittone G, Gullino ML, Spadaro D. Effect of bacterial canker caused by Pseudomonas syringae pv. actinidiae on postharvest quality and rots of kiwifruit ‘Hayward’. Postharvest Biology & Technology. 2016; 1: 119-124. doi: 10.1016/j.postharvbio.2015.11.010

25. Abelleira A, Ares A, Aguin O, Peñalver J, Morente MC, López MM, et al. Detection and characterization of Pseudomonas syringae pv. actinidifoliorum in kiwifruit in Spain. Journal of Applied Microbiology. 2015; 6: 1659-1671. doi: 10.1111/jam.12968

26. Abelleira A, Ares A, Aguín O, Picoaga A, López MM, Mansilla P. Current situation and characterization of Pseudomonas syringae pv. actinidiae on kiwifruit in Galicia (northwest Spain). Plant Pathology. 2014; 3: 691–699. doi: 10.1111/ppa.12125

27. Mccann HC, Eha R, Bertels F, Fiers M, Lu A, Reesgeorge J, et al. Genomic Analysis of the Kiwifruit Pathogen Pseudomonas syringae pv. actinidiae Provides Insight into the Origins of an Emergent Plant Disease. PLoS pathogens. 2013; 9: e1003503. doi: 10.1371/journal.ppat.1003503

28. Vanneste JL, Yu J, Cornish DA, Tanner DJ, Windner R, Chapman JR, et al. Identification, virulence, and distribution of two biovars of Pseudomonas syringae pv. actinidiae in New Zealand. Plant Disease. 2013; 6: 708-719. doi: 10.1094/PDIS-07-12-0700-RE

29. Butler MI, Stockwell PA, Black MA, Day RC, Lamont IL, Poulter RTM. Pseudomonas syringae pv. actinidiae from Recent Outbreaks of Kiwifruit Bacterial Canker Belong to Different Clones That Originated in China. Plos One. 2013; 2: e57464. doi: 10.1371/journal.pone.0057464 PMID: 23555547

30. Liu Y. The bacteriostasis effect of two species of insect antimicrobial peptides crude extract on Pseudomonas syringae pv. actinidiae and the agent control experiments on kiwifruit bacterial canker. Master degree. Thesis, Sichuan Agricultural University. 2016.

31. Chen H. Study on the various detection methods for Pseudomonas syringae pv. actinidiae. Master degree. Thesis, Sichuan Agricultural University. 2017.
