# Supplementary material for: Modeling and mapping the current and future distribution of Pseudomonas syringae pv. actinidiae under climate change in China
Source: PLoS One. 2018 Feb 1;13(2):e0192153. doi: 10.1371/journal.pone.0192153 (PMC5794145; doi:10.1371/journal.pone.0192153)
Supplement: S2 Table — (DOCX) [file pone.0192153.s002.docx]

**S2 Table. List of environmental variables used for this study, with type and measurement unit**

| **Type** | **Code** | **Environmental variables** | **Unit** |
| --- | --- | --- | --- |
| bioclimatic | bio2 | Mean Diurnal Range (Mean of monthly (max temp - min temp) | ℃ |
|  | bio5 | Max Temperature of Warmest Month | ℃ |
|  | bio6 | Min Temperature of Coldest Month | ℃ |
|  | bio7 | Temperature Annual Range (BIO5-BIO6) | ℃ |
|  | bio9 | Mean Temperature of Driest Quarter | ℃ |
|  | bio11 | Mean Temperature of Coldest Quarter | ℃ |
|  | bio12 | Annual Precipitation | mm |
|  | bio14 | Precipitation of Driest Month | mm |
| monthly climatic | prec5, 9, 12 | Precipitation in May, September, December | mm |
|  | tmax2, 4, 9, 10, 11, 12 | Maximum temperature in February, April, September, October, November, December | ℃ |
|  | tmin3, 4, 10, 11 | Minimum temperature in March, April, October, November | ℃ |
|  | tmean5 | Mean temperature in May | ℃ |
