# Supplementary material for: Modeling and mapping the current and future distribution of Pseudomonas syringae pv. actinidiae under climate change in China
Source: PLoS One. 2018 Feb 1;13(2):e0192153. doi: 10.1371/journal.pone.0192153 (PMC5794145; doi:10.1371/journal.pone.0192153)
Supplement: S3 Table — (DOCX) [file pone.0192153.s003.docx]

**S3 Table.** **The** **evaluation criterion of AUC**

| **Range of AUC values** | **Evaluation criterion** |
| --- | --- |
| 0.5≤AUC＜0.6 | Fail |
| 0.6≤AUC＜0.7 | Poor |
| 0.7≤AUC＜0.8 | Fair |
| 0.8≤AUC＜0.9 | Good |
| 0.9≤AUC＜1.0 | Excellent |
